# Error recovery in wearable robotic Co-Grasping: the role of human-led correction

**DOI:** 10.3389/frobt.2025.1598296

**Published:** 2025-10-09

**Authors:** Erin Y. Chang, Wilson O. Torres, Hannah S. Stuart

**Affiliations:** Embodied Dexterity Group, Department Mechanical Engineering, University of California, Berkeley, CA, United States

**Keywords:** wearable robotics, error recovery, co-grasp, cooperation, collaboration, body power, robotic, trust in human-robot interaction

## Abstract

**Introduction:**

Trust in automated systems influences the use and disuse of new technologies. Although recent advances in robotics have improved wearable devices designed to assist in grasping, perfectly reliable systems have yet to be achieved. In this work, we introduce a new strategy for wearable devices called Co-Grasping, where both body power and robotics can contribute to grasping, but the user controls the allocation of the human and robot roles.

**Methods:**

Our implementation of a Co-Grasping device successfully allows the human operator to intervene using body power during simulated robot errors, in order to aid in error recovery and continue performing grasping tasks without drops.

**Results:**

Here, we also show that the presence of recoverable errors lowers trust perception and increases physical engagement behaviors. However, when the robot becomes reliable once again, trust rebounds and most behavioral metrics return to baseline as well.

**Discussion:**

These results indicate that trust in faulty automation can be repaired and that enabling users to assume control over system actuation in response to such faults can prevent errors from negatively affecting overall device function. Facilitating human-led dynamic changes in human and robot role allocation through this Co-Grasping device lays a promising foundation for unique human-robot interactions that promote high performance and where trust can recover quickly, despite existing challenges in developing perfect automated systems.

## 1 Introduction

The development of body-worn robots has become increasingly popular as they aim to augment the abilities of their human wearers. Wearable devices elicit a sense of embodiment in their users, influencing acceptance and use, in addition to their functional benefits (Pazzaglia and Molinari, 2016; Xia et al., 2024). These have been used to assist users who want additional functional assistance with manual activities, often as a result of injury, disability, or other impairments. Robotic devices like prostheses, exoskeletons, and supernumerary limbs have been designed to meet these needs and can provide many benefits like improved grasp force, stability, or dexterity (Huamanchahua et al., 2021; Noronha and Accoto, 2021; Prattichizzo et al., 2021; Lee et al., 2025; Chang et al., 2024a).

While new wearable robotic devices have reached promising accuracy levels above 90% (Hennig et al., 2020; Fougner et al., 2011; Ryser et al., 2017; Li et al., 2019), even 1% grasp inaccuracy can be frustrating and dangerous if dropping an item has associated hazards, like burns from a spilled cup of hot coffee. Small inaccuracies can compound significantly over the course of a day, where an estimated 4,000–7,000 grasps may be performed (Saudabayev et al., 2018; Bullock et al., 2013; Vergara et al., 2014). These errors negatively impact users’ trust in robotic systems (Salem et al., 2015; Desai et al., 2012; Centeio Jorge et al., 2023; Hancock et al., 2011), which is a critical factor in the adoption and continued use of robotic technology (Parasuraman and Riley, 1997; Lee and See, 2004).

In many of these innovative robotic grasping technologies, the human user primarily takes on the role of intent generation, signaling to the automated system what they want to achieve, and then waiting for the robot to process this signal and execute the task. This sequential pathway limits the user’s ongoing involvement, leaving the system fully reliant on the robot’s performance. As a result, any errors made by the robot cannot inherently be recovered, unless the robot is programmed to do so. Robotic errors can thus be frustrating and confusing, so wearable robotic devices that additionally leverage the perceptions and capabilities of the human wearer may be better received by their intended users. Recent work utilizing EEG signals to identify and correct automation errors have shown promise in assisting users with robotic error recovery, however, error classification rates from these signals remain on the order of 10%–20% (Salazar-Gomez et al., 2017; Ferracuti et al., 2022).

A special class of body-worn robotic grasping devices has recently emerged that places body-powered actuation in parallel with robotic actuation, allowing the person and robot to directly alter grasp state in tandem. Usually worn on the hand and wrist, each agent controls separate transmission mechanisms that converge to create the grasp. Exoskeletons have previously used kinematic inversion to map finger and thumb motion onto both the wrist and motor (McPherson et al., 2020; Chang et al., 2022). These devices are unique because they allow task completion even with robot-related issues, like power failures. A tendon-driven supernumerary gripper leverages user wrist strength in extension to apply equal and opposite forces against robotic fingers (Lee et al., 2021; 2025). This device allows users to release grasped objects rapidly without any robotic actuation. In the event of robotic errors or lag, users can still move their respective component toward the now-stationary robotic component, which provides a non-back-drivable resistive force. Thus, it follows that enabling the human operator to productively intervene in the event of an error holds the potential to bridge the gap between imperfect automation and functional resilience of wearable robotic devices.

In the present study, we focus on wrist-driven robotic exoskeletons. By placing body power in parallel with robotic actuation, these wearables allow for dynamic role changes between human and robot during grasping activities. Prior studies programmed robotic components to collaborate with the human user by following the user’s lead and mirroring their movements at all times, like constantly mapping motor motion to wrist motion (Chang et al., 2024a; b). Unlike in collaboration, where the human and robot perform the same roles, agents within these devices can alternatively cooperate. In cooperation, the human and robot perform different or unequal roles (Jarrassé et al., 2012), for example, if the robot moved the fingers while the wrist remained stationary. Depending on the human-robot interaction designed, the two agents may contribute simultaneously, sequentially, or even independently at times. In this work, we seek to observe how the person responds to robotic actuation, whether they choose to collaborate or cooperate. Dynamic changes in role allocation have been studied in external robotic systems (Losey et al., 2018), but not in wrist- and motor-actuated exoskeleton grasping. Here we present what we call a Co-Grasping device, which allows for human-moderated changes between collaboration and cooperation roles.

To the authors’ knowledge, the effect of robot errors on humans has not been studied in wearable robotic Co-Grasp devices where body-powered error recovery is possible. Therefore, we conducted an experiment with a Co-Grasp device programmed to purposefully induce recoverable robotic errors during grasping to:

1. Characterize user behavior and perceptions during robot error situations, and
2. Determine how errors alter trust in the system and user behavior.

Understanding how trust and human behavior change when users have the ability to lead the recovery of robot errors can help improve the way we design effective robots and interactions. To understand these responses, we first present the experimental Co-Grasp robotic device and details of the experimental setup in Section 2. In Section 3, we present the findings of our experiment and discuss the results, limitations, and future work in Sections 4, 5.

## 2 Materials and methods

Wizard-of-Oz methodology is a popular choice in human-robot interaction (HRI) experiments, as it allows complex robotic interactions to be implemented rapidly and with a seamless user experience (Chapa Sirithunge et al., 2018; Rietz et al., 2021; Helgert et al., 2024). The researcher, or “wizard,” remotely controls the robot while monitoring participant interactions with the system; the participant is unaware that a human is controlling the system (Helgert et al., 2024). We implement Wizard-of-Oz control of a custom wearable gripper’s opening and closing in response to verbal input commands from the participant. Participants in this study were told that the robotic system was voice-activated and performed 81 grasps using verbal commands. Between the 27th and 54th grasping activities, we simulated random errors to evaluate user behavior and response before, after, and during robot errors.

Robot errors in wearable grasping devices can take on many forms and typically result from classification errors (Amsüss et al., 2013; Li et al., 2010). These errors can appear as incorrectly predicted actions, such as accidental opening or closing, incomplete action, excessive action, or no action at all. In this work, a single error type was evaluated: an incomplete robotic grasp action. We selected this error type to facilitate human Co-Grasping responses in this initial study, though investigating other error types in the future is recommended.

### 2.1 Wearable robotic device

**FIGURE 1:**
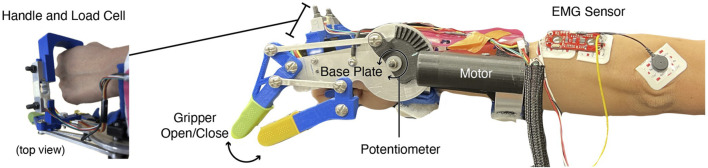
Robotic device used in study. Both robot and operator can open and close the gripper end effector through motor and wrist movement, respectively. User and robot forces are obtained via the load cell in the handle. Wrist movement is measured by a potentiometer located on the interior side of the device base plate. EMG data is collected from the dorsal side of the forearm, with a sensor placed approximately on the primary wrist extensors.

**FIGURE 2:**
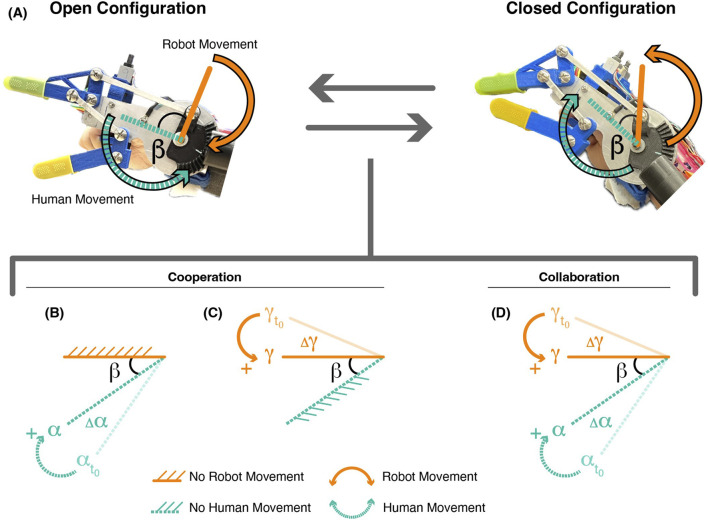
**(A)** Device in the open and closed configuration. β is set by both wrist (α) and motor (γ) position and the gripper can be closed or opened with the movement of one or both. Transition between these two modalities during grasping can occur via cooperation or collaboration. Examples of cooperation behaviors are depicted where **(B)** the human moves the wrist, but the robot does not move the motor, and **(C)** the robot moves the motor, but the human does not move the wrist. In collaboration, **(D)** both the human moves the wrist and the robot moves the motor. Dotted lines indicate elements controlled by the human and solid lines indicate elements controlled by the robot.

The wearable robotic device design shown in Figure 1 is based on the motorized wrist-driven orthoses (MWDO) detailed by McPherson et al. (2020) and Chang et al. (2022). We retain the four-bar linkage mechanism that actuates the MWDO, where robot and human motion contribute to gripper opening and closing (Figure 2A). The planar kinematics of this linkage is described in Chang et al. (2024a). In prior work, device software mapped motor motion directly to wrist movement, constraining the human and robot agents to move simultaneously and in constant collaboration. However, the software implemented on the Co-Grasping device in this work does not map either agent’s movement to the other, instead facilitating a range of interaction types. While collaboration between the human and robot is still possible in this device format, it is no longer required. In Figures 2B–D, we show how motion contributions from the human (Δα) and robot (Δγ) form the interior angle, β , where $\beta =\beta_{t_{0}}-\left(\Delta \alpha +\Delta \gamma \right)$ ; $\Delta \alpha =\alpha -\alpha_{t_{0}}$ , $\Delta \gamma =\gamma -\gamma_{t_{0}}$ , and $t_{0}$ is the time at the start of the grasping phase. The grasping phase begins when the open hand is placed around an object and the first grasping action is initiated. Decreasing β drives the gripper closed, while increasing β drives the gripper open. When the gripper closes around an object, the human and robot must apply opposing static reaction forces to maintain the grip, regardless of the behaviors ( Δα and Δγ ) used to secure it. Note that $\alpha_{t_{0}}$ , $\gamma_{t_{0}}$ , and $\beta_{t_{0}}$ can vary between grasps, depending on how each grasp is initiated. In this study, we initiate grasps such that $\beta_{t0}$ always begins at the same value, as detailed in Section 2.1.1.

Participants have the physical ability to move their wrist as they wish at any time. This ability determines whether they collaborate or cooperate once the robot starts moving. Because the human and robot can move at different times after grasping is initiated, the moments at which the human and robot agents initiate and complete their own grasping actions, i.e., start and stop moving, are defined as $t_{\alpha_{i}}$ , $t_{\alpha_{f}}$ , $t_{\gamma_{i}}$ , and $t_{\gamma_{f}}$ , respectively. In the present work, user voice commands initiate the grasp such that the robot moves to close the gripper immediately (see Section 2.1.1), so we assume $t_{\gamma_{i}}=t_{0}$ . We additionally define the speeds, including magnitude and direction, at which each agent moves at any given moment as $\dot{\alpha }$ and $\dot{\gamma }$ , and the resulting speed $\dot{\beta }$ .

Using these terms, we define the possible grasping behaviors. We consider grasping as an agonistic task, where both agents contribute only to the improvement of the shared activity (Jarrassé et al., 2012), such that grasping occurs when $\dot{\beta }<0$ and is constrained to $\dot{\alpha },\dot{\gamma }\geq 0$ . The human and robot exhibit collaborative grasping whenever they move concurrently to reduce β , where $\dot{\alpha }>0$ and $\dot{\gamma }>0$ . The matching of these movements by both agents in collaboration is shown in Figure 2D. Alternatively, the grasping behavior is cooperative if either $\dot{\alpha }=0$ or $\dot{\gamma }=0$ . Figure 2B shows the participant cooperating with the robot by letting the robot move entirely while the wrist remains stationary $\left(\dot{\alpha }=0\right)$ and Figure 2C shows the opposite. In a single grasp, both cooperation and collaboration can occur at different times. For example, if the robot begins to move before the human $\left(t_{\gamma_{i}}<t_{\alpha_{i}}\right)$ , the grasp starts in cooperation. Then, if the robot’s motion finishes after the human’s motion $\left(t_{\alpha_{i}}<t_{\gamma_{f}}\right)$ , a collaborative grasp phase can occur. The grasp may later return to cooperation if the agents do not complete their movements at the same time $\left(t_{\alpha_{f}}\neq t_{\gamma_{f}}\right)$ .

Measuring how a person chooses to interact with the system when both collaboration and cooperation are possible, however, has yet to be quantified. Sensors integrated into the current test setup allow for this observation. Within the Co-Grasping device, a soft rotary potentiometer (Spectra Symbol, Salt Lake City, Utah, USA) measures the human’s wrist position (α) and an encoder measures the robot’s motor position (γ) . These sensors quantify the physical participation of each agent through their relative movements and effect on β . We also uniquely augment the original design of the MWDO to additionally measure forces exerted by and on the user. Unlike the original MWDO designs, which actuate the user’s own fingers, the linkage structure in the Co-Grasp device actuates a two-pronged artificial gripper. The user grasps a handle attached perpendicularly to the base plate with a 5 kg load cell (SparkFun, Niwot, Colorado, USA) to measure the amount of force exerted between the operator and the device (Figure 1). The user feels the reaction forces needed to maintain a successful grasp through this handle, providing inherent feedback on the state of the system that is typically not provided by motorized components of other robotic grasping technologies. When forces increase during grasping, relative motion of the human or motor indicates which agent leads force application. Because wrist muscular exertion is necessary to maintain a grasp, we estimate resulting muscle activity using a Myoware v1.0 electromyography (EMG) sensor (Advancer Technologies, Raleigh, North Carolina, USA) on the wrist extensors. These EMG measurements add a physiological perspective to the measured behavioral changes.

#### 2.1.1 Control and actuation

**FIGURE 3:**
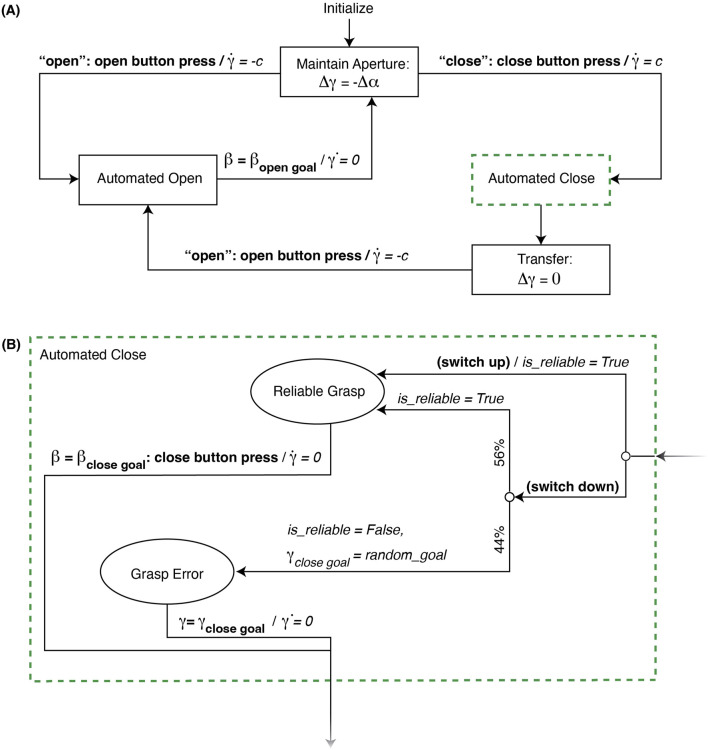
Device state machine **(A)** overview and **(B)** detailed grasping logic. When the switch is down in **(B)**, the probability of Grasp Error is 44% and Reliable Grasp is 56%. Following Reliable Grasp, $\beta =\beta_{\mathrm{closegoal}}$ is observed by the researcher. Legend: Rectangles outline device states, while transitioning arrows are labeled with events, conditions, and responses needed to move between control states. Bold text in quotes are user commands and bold text without quotes are researcher actions or observations. Bold text in parentheses denotes conditions that must be met and *italic text* denote robot responses. A slash (/) separates the event or condition from the subsequent response.

The implemented finite state machine (FSM), pictured in Figure 3, represents the software used to control the robotic agent throughout the study. Participants primarily interacted with the system via voice commands, using the key words “*open*” and “*close*” to indicate to the robot which actions to perform. Unbeknownst to the participants, a researcher manually controlled these “automated” aspects in response to voice commands using buttons to trigger appropriate events. We deliberately implemented a Wizard-of-Oz setup to ensure complete researcher control over the robotic elements of the system and prevent unintended robotic error behaviors that might arise from computerized voice recognition.

To initialize each trial, participants were instructed to hold their wrist steady and in a neutral posture for the duration of a robot calibration phase and say “*calibrate*” when they were ready for this phase to begin. In response to this voice command, the researcher used buttons to close the gripper fingers, then transitioned the device control to the *Maintain Aperture* state in Figure 3A. In this state, the user could position their wrist as desired, while the robot tracked their wrist position using the potentiometer to maintain the gripper’s current aperture. This relationship was defined as: Δγ=−Δα . The motor was controlled to achieve Δγ+Δα=0 using a cascaded hand-tuned controller consisting of a proportional position outer loop and a proportional-integral velocity inner loop. This *Maintain Aperture* state was adapted from Chang et al. (2024b), allowing the user to position their upper limb comfortably prior to grasp or release by freely moving their wrist independently of gripper orientation.

Prior to grasping, the participant said “*open*” and the researcher pressed the open button to transition the system to the *Automated Open* state, where the motor moved at a constant speed $\left(\dot{\gamma }=-c\right)$ using a proportional feedback controller, such that Δγ became increasingly negative, increasing β . Once β reached $\beta_{\mathrm{opengoal}}$ , measured to be 95°, the system automatically returned to the *Maintain Aperture* state, once again allowing free movement of the wrist so the user could choose a comfortable grasping posture. The user then positioned the open gripper around the target object and said “*close*” to initiate the grasp. At grasp initiation, $\beta =\beta_{t0}=\beta_{\mathrm{opengoal}}$ . The researcher then pressed the close button once to transition the device into the *Automated Close* state, where the robot began moving the motor at a constant speed $\left(\dot{\gamma }=c\right)$ , such that Δγ became increasingly positive, decreasing β .

The direction of a control switch determined the subsequent automated close behavior shown in Figure 3B. When the control switch was up, the system facilitated a *Reliable Grasp*, where the device simulated “ideal” or successful grasping by halting motor movement only when the researcher visually determined $\beta =\beta_{\mathrm{closegoal}}$ , which was approximately 65° and when the object was secured within the gripper. Upon this observation, the researcher pressed the same close button again, thus transitioning to the *Transfer* state. Alternatively, when the control switch was down, the system pseudo-randomly facilitated a *Grasp Error*, 44% of the time. In these instances, $\gamma_{\mathrm{closegoal}}$ was pseudorandomly defined as a motor contribution angle varying between 6–18°, or 20%–60% of the γ needed to securely grasp the test object, resulting in insufficient gripper closure $\left(\beta >\beta_{\mathrm{closegoal}}\right)$ . Despite this unsuccessful grasp, the system automatically entered the *Transfer* state after prematurely stopping robot motion. Completing grasps successfully after a *Grasp Error* required the participant to move their wrist (+Δα) to actuate the device and finish closing the gripper $\left(\beta =\beta_{\mathrm{closegoal}}\right)$ . This error type was selected to highlight a unique attribute of this class of wearable robotic devices, namely, the potential for error recovery by the human operator. In the *Transfer* state, the robot no longer moved the motor (Δγ=0) until the user said “*open*” again to initiate the open button press by the researcher, which would command the motor to move the gripper once again in the *Automated Open* state.

### 2.2 Experimental protocol

Participants grasped and released a set of test objects between two sets of elevated platforms. Nine right-handed individuals were recruited from the University of California, Berkeley (UCB), with a mean age of 22.56 ± 3 years. Six participants identified as female and three as male. Three participants ethnically identified as Hispanic/Latinx. Four participants racially identified as Asian, four as White, one did not provide their race. All work was performed under the UCB Institutional Review Board protocol #2020-02-12983.

#### 2.2.1 Setup

**FIGURE 4:**
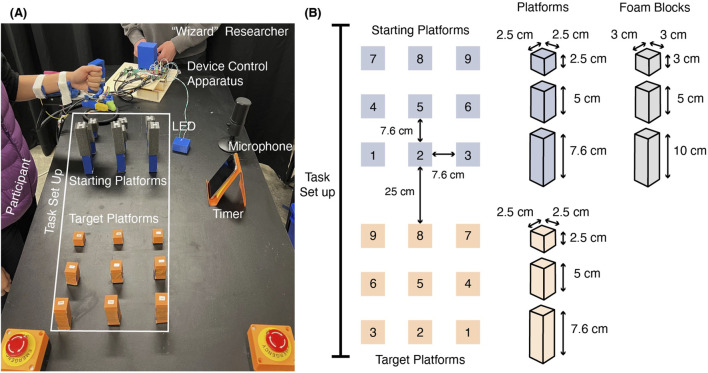
Workspace setup including **(A)** the relative placements of the participant wearing the device, the researcher operating the robot at the device control interface, indicator LED, microphone, and timer, **(B)** dimensions of the task set up, platforms, and foam blocks.

Figure 4 depicts the experimental workspace. Participants stood on one side of the table while wearing the device, while a researcher stood on the adjacent side by the device control apparatus (Figure 4A). The table height was adjusted so that it always sat 10 cm below the participant’s elbow joint. Two sets of platforms were arranged in front of the participant. Subjects were tasked with moving foam blocks in ascending number order from each starting platform to the corresponding target platform with the same number. Platform surfaces measured 2.5 cm × 2.5 cm with heights of 2.5, 5, or 7.6 cm, and each group of starting and target platforms was arranged in front of the participant so that platforms were placed in ascending height order from left to right (Figure 4B). Each set of platforms was labeled 1–9. Foam blocks of three different heights (3, 5, and 10 cm) were placed on top of the starting platforms. The shortest blocks were placed on top of the shortest starting platforms, and the tallest blocks were placed on top of the tallest starting platforms. The order of both the platform numbers and heights was designed to reduce the risk of knocking nearby blocks during grasp and release. However, the close proximity between targets and small platform surface areas were selected to encourage grasping precision.

To add a sense of urgency, a visible timer was also placed on the table and participants were instructed to perform tasks as quickly as possible without dropping any blocks. Researchers informed the participant that any dropped blocks would incur a time penalty, however, this time penalty was not included in analysis. To support the illusion of true automation, participants were told that the microphone placed on the table in front of them was listening for their voice commands. We also placed an LED-based light indicator on the table to visually communicate to the user when the robot had completed its grasping action. The LED illuminated when the system entered the *Transfer* state.

#### 2.2.2 Procedure

At the beginning of the experiment, the researcher first introduced the robot to the participant by demonstrating the robot’s grasping ability, the option to control the gripper with wrist movement, and the voice commands needed to operate the robotic system. The researcher also demonstrated an error, informing the participant that “this device is a prototype and it is possible that it may malfunction from time to time.” During this demonstration, the researcher maintained a constant wrist position (α) until the robot stopped moving, then extended their wrist to complete the grasp. Since participants were unfamiliar with this device, demonstrating this recovery tactic ensured that they knew how to intervene if desired. To measure initial trust in the system after these interactions, participants completed the 14-item Trust Perception Scale-HRI (TPS), developed to subjectively measure trust perception from 0% to 100% during HRI studies and across a variety of robotic domains (Schaefer, 2016).

The device was then fitted onto the participant’s left upper limb such that their wrist joint aligned with the device’s wrist joint when holding the device handle. The EMG sensor picture in Figure 1 was applied to the dorsal side of the participant’s forearm, about 2.5 cm from the elbow and along the muscle belly of the extensor carpi radialis. Researchers located this region by palpation while the participant extended their wrist. EMG sensor gain was tuned such that muscle activation did not saturate the signal. In one subject, the EMG sensor failed to properly record data for some trials and was thus omitted from their dataset for analysis. For this subject, only data from the potentiometer and load cell were collected. The potentiometer, load cell, and EMG sensor all recorded measurements at 25 Hz.

Device calibration (described in Section 2.1.1) was performed prior to the start of every trial. One trial was defined as when the participant moved the set of nine foam blocks from the starting platforms to the target platforms. The grasp, transfer, and release of each foam block made up a subtask, such that nine subtasks made up one trial. Each subject performed nine trials, which were divided into *Pre*-error, *Error*, and *Post*-error conditions. They performed three trials in each condition before proceeding to the next. During the *Pre*-error and *Post*-error trials, user commands to the robot resulted in automated *Reliable Grasps*. During each *Error* trial, the robot generated automated *Grasp Errors* during four pseudorandom subtasks per trial.

After each trial, participants completed the TPS while considering their entire experience with the device since the beginning of the study, so we could measure changes in overall trust over time. They also completed the NASA Task Load Index (NASA-TLX), which measures their perceptions of different workload aspects (Hart, 1986); for this survey, they considered only their experience during the preceding trial, so we could measure changes in task difficulty during different conditions. In total, they completed the TPS ten times (once following the researcher demonstration and once following each of the nine trials) and the NASA-TLX nine times throughout the study (following each trial).

If a foam block fell during a subtask, participants were instructed to leave it behind and proceed to the next subtask. A “drop” was defined as any time a foam block fell from the gripper and did not land on the target platform, between the subject’s “close” and “open” commands. On occasion, foam blocks fell from the target platforms due to environmental interferences, like the participant bumping onto the block with their forearm/device or accidentally moving the table. These were quickly replaced by a researcher to prevent time delays and were not recorded as drops.

### 2.3 Data analysis

Wrist angle, force, and EMG sensor data from each subtask was parsed into 3 segments: *robot grasp*, *object transfer*, and *robot release*. The *robot grasp* segment began when the robot began closing the gripper (start of *Automated Close* state in Figure 3) and ended when the robot stopped closing the gripper (start of *Transfer* state in Figure 3). *Object transfer* took place immediately after the robot finished its closing action, when the human had to lift and transfer the object to the next location, lasting the duration of the *Transfer* state in Figure 3. Finally, *robot release* began when the robot began opening the gripper and ended when it stopped opening the gripper, lasting the duration of the *Automated Open* state of Figure 3. During initial examination of the data, subjects typically remained passive during the *robot grasp* and *robot release* phases of each subtask. Participants only began interacting with the system during the *object transfer* phase, where they took on either a static and resistive role to hold the gripper steady while moving the object, or enacted wrist motion to recover from the robot’s error before moving the object. Therefore, we focused sensor-based analysis efforts on data from the *object transfer* segment.

In post-processing of the sensor data, a moving average filter with a window size of 5 data points (0.2 s) was initially applied to smooth sensor signals prior to subsequent calculations. Each signal was normalized to the lowest value of the current segment, such that the resulting processed time-series data represented relative changes in signal specific to the current subtask, instead of absolute changes in signal. Peaks were defined as the maximum value of the normalized signal. Additionally, since the duration of *object transfer* segments varied across subtasks and participants, we scaled timestamps to a common range, in order to maintain temporal data patterns independent of absolute time. Instead, each timestamp, s , was represented as a proportion of the total duration of the *object transfer* phase, $s_{\mathrm{total}}$ , such that normalized time $=\left(s/s_{\mathrm{total}}\right)*100$ .

Objective metrics, taken from data collected during trials, included the following:

1. Total Wrist Motion: the area under the curve (AUC) of the normalized potentiometer data, in units of degrees * $s/s_{\mathrm{total}}$ .
2. Total Force: the AUC of the normalized load cell data, in units of kg * $s/s_{\mathrm{total}}$ .
3. Peak Normalized Force: the largest value in normalized load cell data, in *kg.*
4. Relative Time to Peak Normalized Force: the time at which the largest value in normalized load cell data occurs, in $s/s_{\mathrm{total}}$ .
5. Initial Force Activity: the slope between normalized load cell readings at 10% relative time and 0% relative time, in units of *kg/* $\left(s/s_{\mathrm{total}}\right)$ . This measures the participants’ immediate reactions to experiencing an error, via their applied force to the system.
6. Total Muscle Activity: the AUC of the normalized EMG data, in units of mV * $s/s_{\mathrm{total}}$ .
7. Peak Normalized Muscle Activity: the largest value in normalized EMG data, in *mV.*
8. Relative Time to Peak Normalized Muscle Activity: the time at which the largest value in normalized EMG data occurs, in $s/s_{\mathrm{total}}$ .
9. Sustained Peak Normalized Muscle Activity: the AUC of +/− 5% relative time window around the Peak Normalized Muscle Activity, in units of mV * $s/s_{\mathrm{total}}$ . This measures whether maximum muscle activity is more of an impulse or a sustained contraction.
10. Task Completion Time: time it took to complete a single trial comprising nine grasping subtasks, in *seconds.*
11. Object Drops: average number of object drops in a trial.

Subjective metrics taken from surveys in between trials included the following:

12. Trust: participants’ self-reported trust levels out of 100%, as measured by the Trust Perception Scale (Schaefer, 2016).
13. Workload: participants’ self-reported perceived workload out of 100, as measured by the NASA Task Load Index (Hart, 1986).

For objective metrics 1-9, each subject’s dataset was averaged to obtain a single value for each condition during the *object transfer* segment. From the *Error* condition trials, only data from the 12 subtasks where the robot error occurred were pooled together, excluding the remaining 15 subtasks during these trials where the robot performed normally. Objective metrics 10–11 and subjective metrics 12–13 were recorded for each trial and averaged by condition for each subject.

An omnibus Friedman’s test was performed to determine if the robot behavior condition significantly affected each metric, while accounting for individual subject differences. If the result of the omnibus test was significant (p < 0.05), a Nemenyi *post hoc* test was performed to determine which conditions differed from each other, while accounting for both subject-level differences and multiple comparisons. Paired comparisons were considered statistically significant when adjusted p-values from Nemenyi’s test were < 0.05. For specific comparisons between two trials, p-values were calculated using the Wilcoxon Signed-Rank test and considered statistically significant when p < 0.05.

## 3 Results

Prior to donning the device, average participant trust in the system was 69.21 +/− 4.26%, however, after donning and using the device for the first time, mean trust rose to 85.85 +/− 10.65%, with every individual participant’s score increasing. Because use increased trust, unfamiliarity with the system may have negatively influenced participants’ perception prior to use.

### 3.1 Error characterization

**FIGURE 5:**
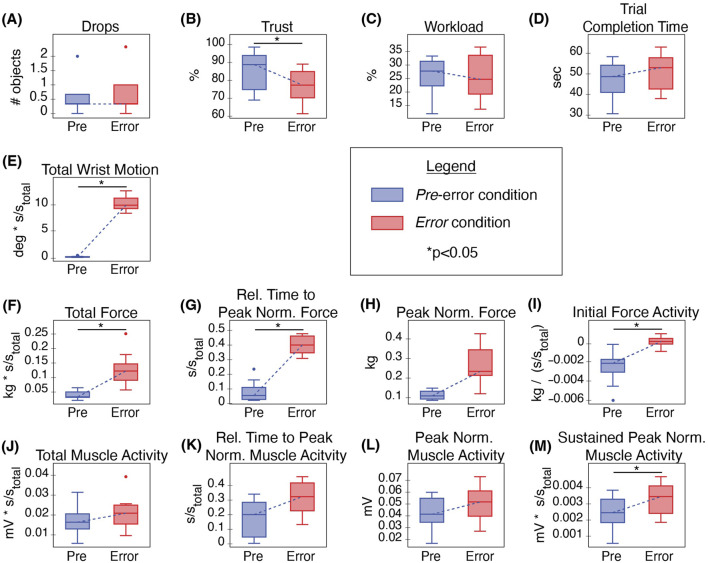
Comparison of *Pre*-error and *Error* conditions for **(A)** average object drops per trial, **(B)** trust, **(C)**) workload, **(D)** trial completion time, **(E)** total wrist motion, **(F)** total force, **(G)** relative time to peak normalized force, **(H)** peak normalized force, **(I)** initial force activity, **(J)** total muscle activity, **(K)** relative time to peak normalized muscle activity, **(L)** peak normalized muscle activity, and **(M)** sustained peak normalized muscle activity.

Participants successfully recovered from most robot grasp errors, shown by one or fewer object drops per trial on average and no statistically significant difference in average drops between the *Pre*-error and *Error* trials (Figure 5A). Despite frequently recovering from these errors, errors impacted participants’ perceptions of the robot; *Error* condition trust levels dropped significantly from the *Pre*-error baseline (p = 0.003, Q = 3.30), shown in Figure 5B. Perceived workload and trial completion time, however, did not significantly change (Figures 5C,D).

Errors significantly affected many user behaviors. During the *Pre*-error condition, we consistently observed robot-led cooperation, with no human wrist motion detected at all (Figure 5E), meaning $\dot{\alpha }=0$ . When errors occurred, total wrist motion significantly increased (p = 0.01, Q = 2.83), indicating a change to human-led cooperation with the robot ( $\dot{\alpha }>0$ and $\dot{\gamma }=0$ ). When participants did move their wrist, their reaction time occurred around 20%–30% of normalized time after robot movement was completed. We did not observe any instances of collaboration, where human and robot movements overlapped.

Many exertion-related behaviors were also affected by errors. Total force, relative time to peak normalized force, peak normalized force, and initial force activity all showed increased during error trials, shown in Figures 5F–I. Furthermore, the increases in total force (p = 0.003, Q = 3.30), relative time to peak normalized force (p = 0.0001, Q = 4.01), and initial force activity (p = 0.003, Q = 3.30) were statistically significant (Figures 5F,G,I). On the other hand, most muscle activity metrics were not significantly affected by the errors (Figures 5J–L), with the exception of sustained peak normalized muscle activity (p = 0.008, Q = 3.00), shown in Figure 5M.

During the *Pre*-error condition, subjects chose to cooperate with the robot, holding their wrist stationary and allowing the robot to lead by taking complete control of actuating the gripper closed. In the *Error* condition, they also initially opted for robot-led cooperation, however, during the 44% of instances where the robot failed to accomplish the task alone, they utilized body power to reverse the roles and lead the remainder of the gripper actuation. This human-led motion against a stationary robot agent applying passive resistive force allowed the human to successfully recover the task from robot error.

### 3.2 Post-error effects

**FIGURE 6:**
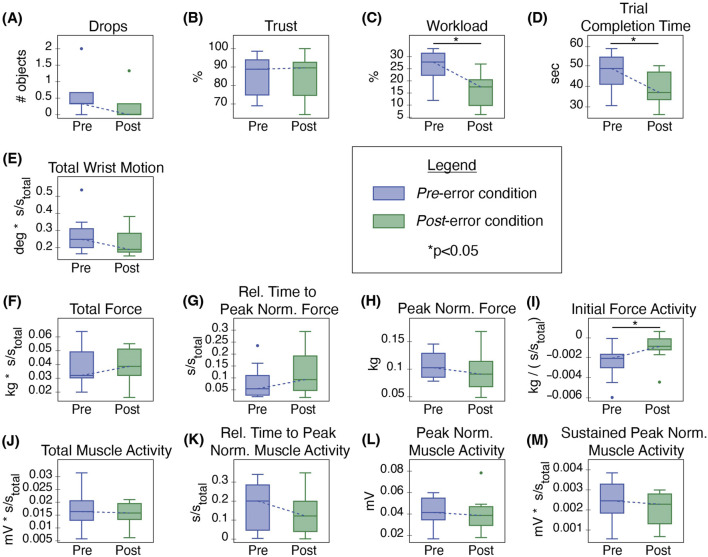
Comparison of *Pre*-error and *Post*-error conditions for **(A)** average object drops per trial, **(B)** trust, **(C)** workload, **(D)** trial completion time, **(E)** total wrist motion, **(F)** total force, **(G)** relative time to peak normalized force, **(H)** peak normalized force, **(I)** initial force activity, **(J)** total muscle activity, **(K)** relative time to peak normalized muscle activity, **(L)** peak normalized muscle activity, and **(M)** sustained peak normalized muscle activity.

Following errors, in the final *Post*-error condition, subjects reverted to constantly utilizing robot-led cooperation, without actively moving their wrists. Participants dropped very few objects (Figure 6A), and this number did not vary significantly. When robot behavior returned to normal in the *Post*-error trials, trust also returned to the original baseline levels, pictured in Figure 6B. No significant difference between trust levels was observed in the *Pre*-error and *Post*-error conditions.

Most behavior metrics paralleled trust and returned to their *Pre*-error baselines, shown by similar value ranges between the two conditions and a lack of statistical significance in the comparison. Wrist motion returned to the zero motion baseline (Figure 6E). Total force, relative time to peak normalized force, peak normalized force, and sustained peak normalized muscle activity also returned to similar *Pre*-error baseline measurements (Figures 6F–H,M).

Three metrics did not return to their baseline levels (Figures 6J–L). As pictured in Figure 6I, initial force activity showed a statistically significant increase between the *Pre*- and *Post*-error conditions (p = 0.048, Q = 2.36). Additionally, users reported significantly lower workloads (p = 0.006, Q = 3.06) and completed trials significantly faster (p = 0.04, Q = 2.47) toward the end of the study, compared to the beginning of the study (Figures 6C,D). A comparison of the first and last trials alone for these three metrics showed the same statistically significant trends. A similar comparison of the other behavioral metrics did not show statistically significant differences, except for sustained peak normalized muscle activity (p = 0.008, z = 2.52).

## 4 Discussion

### 4.1 Error characterization

Changes in wrist motion clearly indicated increased human engagement during robot errors. At the beginning of the experiment, participants were reminded that they could move their wrist at any time to contribute to the grasping action, and we initially expected that they might collaborate with the robot by moving their wrist at the same time to close the gripper faster. Despite the added pressure of timed trials and experiences with robot failures, none of the participants engaged their wrist during the *robot grasp* phase, instead, waiting for the robot to fully complete its action before choosing whether or not to move the wrist. As such, when the robot performed grasps successfully on its own, users chose to cooperate by providing stationary resistance for the system and did not move their wrists, but a short period after the robot errors occurred, they successfully compensated for incomplete grasps by taking the cooperative lead and moving their wrist to finish closing the gripper. Although the exact cause for this behavior cannot be determined here, possible influencing factors could include prioritization of reduced workloads or hesitation to interact with the system during robotic actions, warranting further investigation of these collaborative and cooperative human-robot behaviors in the future.

Participants engaged more with the system, through wrist extension, but trusted it less when the robot exhibited errors. Reductions in trust observed in this work align with findings of other studies (Esterwood and Robert Jr, 2023; Schaefer, 2016; Esterwood and Robert, 2021) indicating the negative influence of robot errors on trust. Additionally, our results showed that trust was reduced even when the user could correct the robot’s error and still successfully complete their task goals. Similar perceived workloads, trial completion times, and object drops further indicated that errors did not significantly affect performance, despite the notable changes in trust during this same time period. This suggests that the overall performance of the wearable system did not correspond to the user’s trust in the system. It is therefore important to consider additional human-centric variables beyond device performance alone when evaluating new wearable technologies to reduce the risk of overlooking critical human factors that could impact device adoption and desirability (Motti and Caine, 2014).

The compensatory wrist movements after the robot had stopped moving caused the human operator to exert higher forces onto the device to correct for the robot’s mistake. In wearable devices where the applied forces from components controlled by the human and the robot are directed toward each other, notable human-applied force changes are an important consideration in the physical design of the system. The device structure must be able to withstand potentially increased forces during errors, which can influence material and electronic component selection. Related to user-applied force, extensor muscle engagement also increased slightly during robot error for all participants. However, these changes were not found to be statistically significant. Closer examination of the EMG signal indicated that muscle activity patterns depended more on the subtask (block position) than the robot condition. Since the positions of the forearm and wrist orient the hand, it is expected that grasping and moving objects from different locations would require varied upper limb orientations.

### 4.2 Post-error effects

Despite insignificant changes in task completion (drops, workload, time) during the error cases, trust in our Co-Grasping device significantly declined when users faced these robot errors. Nonetheless, we found that trust was rebuilt to the original levels after the recoverable errors had ceased. This trust recovery ability supports the continued study of these devices with the potential to foster viable human-robot interactions that sustain human engagement. Similarly, findings from another study of unexpected errors in robotic prosthetic grasping showed that trust declined during operation of the system with errors, and increased back to baseline levels in subsequent trials where robotic behavior returned to normal (Abd et al., 2019). These two studied scenarios indicate that the negative impact of these error types on trust is not permanent. Subsequent studies on the effect of errors within wearable robotic grasping systems should therefore continue evaluating the impacts of other error types, like catastrophic errors (ex. if the robot prematurely re-opens the gripper), and longer interaction times (ex. frequent errors over the course of a day). Identifying potential and foreseeable problems that do not permanently damage users’ trust can be used to guide future human-robot interactive designs that are both effective and desirable. In addition to trust recovery, most behavior patterns returned to baseline, suggesting that users’ perceptions correctly matched their physical responses.

Lasting changes were observed in three metrics. Of the sensor-measured behaviors, the initial force activity in the *Post*-error condition remained significantly different than that in the *Pre*-error condition. These trends in initial force activity suggest that immediate behavioral reactions, whether due to learning effects or the unexpectedness of the robot error, should be investigated in the future. One perception metric and one performance metric did not return to baseline either: subjects reported significantly lower workloads and completed trials faster following errors than prior to errors, implying a learning effect. The tasks became easier for the users over time, and they began to complete the tasks more quickly. However, this learning trend was not observed in most behavioral metrics.

### 4.3 Additional limitations and future work

Wizard-of-Oz studies allow the creation of complex robotic interactions with less complex robots. Nevertheless, they can unintentionally influence behavior if users realize the robot is not autonomous. In our study, most participants seemed convinced of the robot’s voice-activated feature, evidenced by participants speaking louder or getting closer to the microphone during errors, as they believed some errors resulted from their lack of vocal accuracy. Additionally, participants expressed positive sentiments regarding the idea of a voice-activated robot. Future work should include post-study surveys to confirm these perceptions of complete automation.

We additionally did not collect any internal perception information of behavioral changes, like whether users believed they were altering their behavior or if their internal mood changed based on the robot conditions. Including these data points in subsequent works should be considered to obtain a more nuanced understanding of how users respond to robot error. Other sensory modalities such as EEG could be useful in exploring objective cognitive reactions to such errors and anchor other time-based physical reactions like those measured in this work. Future studies should also look to expand the demographic pool of participants to increase generalizability.

Researchers initially demonstrated the error and recovery method to the user in this study. Although we do not believe that this demonstration changed robotic perception based on increased trust after initial device-worn trials, future work should include unseen errors and lengthen the time of the study to allow participants to fully discover device functionality and potential robotic flaws, as well as how to organically recover from them.

Finally, this work presents an initial investigation into the human-led recovery and response to robotic error, however, many types of robotic errors may arise beyond the one evaluated here. We suspect that human responses may change with respect to different types of errors, particularly when considering the context of such errors. Studying other error types and varying the situations in which they arise will be very informative in the design and adoption of new wearable robotic tools.

## 5 Conclusion

We presented a strategy for worn robotic- and body-powered devices called Co-Grasping that allows both human and robot agents to contribute to grasping in parallel in either collaboration or cooperation. This approach uniquely positions the user to determine how to allocate roles and when to dynamically switch between them. The human user can rely on the robot to perform grasping tasks or intervene using body-powered wrist motion. When robots make errors, this relationship enables a new role for human users: the ability to recover from these errors. Through a human-subject experiment with simulated robotic errors via a custom wearable testbed, we found that the Co-Grasping device successfully enabled users to lead the recovery of robot errors, and that users chose to respond in cooperation instead of collaboration.

Specifically, we found that grasp errors changed user behavior and trust perception. We characterized responses to recoverable robot errors by: change in role allocation from robot-led to human-led cooperation, reductions in trust, and increases in wrist movement, force, and sustained wrist extensor muscle activity. While metrics of engagement, like wrist movement, increased with errors, functional performance and perceived workload did not change significantly, indicating the effectiveness of including the body-powered pathway in robotic wearables. The decline in trust, however, was not alleviated with body power during errors, emphasizing the importance of measuring and studying human factors to guide development beyond the feasibility of new solutions alone. Collecting both subjective and objective measures of performance and perception provides a more comprehensive understanding of the human-robot-task interaction than one type alone. After users experienced errors, most measured parameters returned to baseline once the robot behaved ideally again. These findings show the potential benefits of worn robotic devices that facilitate new human-robot interactions with dynamic role allocation, which can be robust in human perception and behavior against robot errors.

## Data Availability

The raw data supporting the conclusions of this article will be made available by the authors, without undue reservation.
